# The pentose phosphate pathway mediates hyperoxia-induced lung vascular dysgenesis and alveolar simplification in neonates

**DOI:** 10.1172/jci.insight.137594

**Published:** 2021-03-08

**Authors:** Jiannan Gong, Zihang Feng, Abigail L. Peterson, Jennifer F. Carr, Xuexin Lu, Haifeng Zhao, Xiangming Ji, You-Yang Zhao, Monique E. De Paepe, Phyllis A. Dennery, Hongwei Yao

**Affiliations:** 1 Department of Molecular Biology, Cell Biology & Biochemistry, Division of Biology and Medicine, Brown University, Providence, Rhode Island, USA.; 2 Department of Respiratory and Critical Care Medicine, Second Hospital of Shanxi Medical University, Taiyuan, Shanxi, China.; 3 Department of Nutrition, Byrdine F. Lewis School of Nursing and Health Professions, Georgia State University, Atlanta, Georgia, USA.; 4 Program for Lung and Vascular Biology, Stanley Manne Children’s Research Institute, Ann & Robert H. Lurie Children’s Hospital of Chicago, Chicago, Illinois, USA.; 5 Departments of Pediatrics (Critical Care Division), Pharmacology, and Medicine (Pulmonary and Critical Care Medicine), Northwestern University Feinberg School of Medicine, Chicago, Illinois, USA.; 6 Department of Pathology, Women and Infants Hospital, Providence, Rhode Island, USA.; 7 Department of Pediatrics, Warren Alpert Medical School of Brown University, Providence, Rhode Island, USA.

**Keywords:** Pulmonology, Glucose metabolism

## Abstract

Dysmorphic pulmonary vascular growth and abnormal endothelial cell (EC) proliferation are paradoxically observed in premature infants with bronchopulmonary dysplasia (BPD), despite vascular pruning. The pentose phosphate pathway (PPP), a metabolic pathway parallel to glycolysis, generates NADPH as a reducing equivalent and ribose 5-phosphate for nucleotide synthesis. It is unknown whether hyperoxia, a known mediator of BPD in rodent models, alters glycolysis and the PPP in lung ECs. We hypothesized that hyperoxia increases glycolysis and the PPP, resulting in abnormal EC proliferation and dysmorphic angiogenesis in neonatal mice. To test this hypothesis, lung ECs and newborn mice were exposed to hyperoxia and allowed to recover in air. Hyperoxia increased glycolysis and the PPP. Increased PPP, but not glycolysis, caused hyperoxia-induced abnormal EC proliferation. Blocking the PPP reduced hyperoxia-induced glucose–derived deoxynucleotide synthesis in cultured ECs. In neonatal mice, hyperoxia-induced abnormal EC proliferation, dysmorphic angiogenesis, and alveolar simplification were augmented by nanoparticle-mediated endothelial overexpression of phosphogluconate dehydrogenase, the second enzyme in the PPP. These effects were attenuated by inhibitors of the PPP. Neonatal hyperoxia augments the PPP, causing abnormal lung EC proliferation, dysmorphic vascular development, and alveolar simplification. These observations provide mechanisms and potential metabolic targets to prevent BPD-associated vascular dysgenesis.

## Graphical abstract

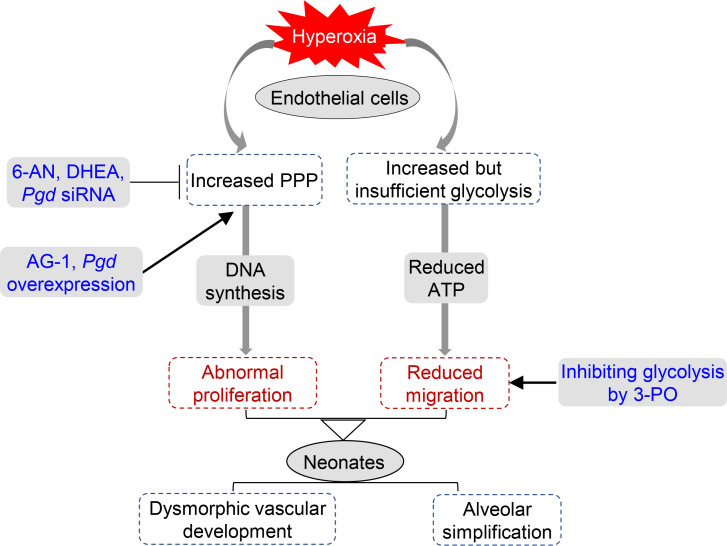

## Introduction

Babies born prematurely have underdeveloped lungs that do not facilitate proper gas exchange; therefore, they need respiratory support, including supplemental oxygen and/or mechanical ventilation, as lifesaving measures. Unfortunately, these therapies can cause bronchopulmonary dysplasia (BPD), a chronic lung disease, which affects 10,000–15,000 premature infants annually in the USA. Although most BPD survivors eventually are weaned off oxygen, they may show evidence of persistent pulmonary injury and lung function decline as adolescents and adults (1, 2). Lung pathology of BPD is characterized by the disruption of alveolar and microvascular development, which results in defective gas exchange. Pulmonary microvascular arrest plays an important role in the pathogenesis of this disease, as the pulmonary vascular bed is essential for alveolar formation during the late stages of lung development (3–7). However, using stereological volumetry approaches, we and others have shown that the pulmonary microvasculature expands in proportion to the growth of the air exchanging parenchyma in ventilated premature infants or premature infants with BPD (8, 9). The expansion of the pulmonary microvasculature is associated with increased proliferation of lung endothelial cells (ECs) (8). Furthermore, these capillaries locate in the lung interstitium and do not align beneath the airway epithelium, thus retaining the primitive vascular pattern of a double-layered capillary network, all of which leads to ineffective gas exchange (8, 9). The mechanisms underlying these abnormally shaped and malpositioned vessels in BPD are not known.

ECs are highly glycolytic, generating more than 80% of their ATP through this pathway (10, 11). Glycolysis is essential for EC proliferation and vessel sprouting, since glycolytic inhibition by genetic ablation of 6-phosphofructo-2-kinase/fructose-2,6-bisphosphatase isoform 3 (PFKFB3) suppresses EC proliferation and angiogenesis (10, 12). The pentose phosphate pathway (PPP), a metabolic pathway parallel to glycolysis, generates NADPH as a reducing equivalent and ribose 5-phosphate for nucleotide synthesis. During early postnatal life, infants born prematurely are at high risk of altered glucose homeostasis, which may have long-term effects on their growth and development (13). A previous study has shown that hyperoxic exposure, a known mediator of BPD in rodent models, reduces glycolytic capacity and reserve in a mouse lung epithelial cell line (14). It is unclear whether in vivo hyperoxic exposure alters glycolysis or the PPP in lung ECs, leading to abnormal EC proliferation and dysmorphic angiogenesis. We hypothesized that hyperoxic exposure augments glycolysis and the PPP in lung ECs, leading to abnormal EC proliferation, dysmorphic vascular development, and lung injury. To test this hypothesis, we exposed newborn mice and lung ECs isolated from neonatal mice to hyperoxia followed by normoxia, since neonatal hyperoxia has long-term and persistent effects on lung function and alveolar simplification (15). We then evaluated glycolysis and the PPP and determined their respective roles in hyperoxia-induced abnormal EC proliferation and subsequent lung injury in neonatal mice.

## Results

### Hyperoxic exposure increases glycolysis in cultured lung ECs.

**Figure 1:**
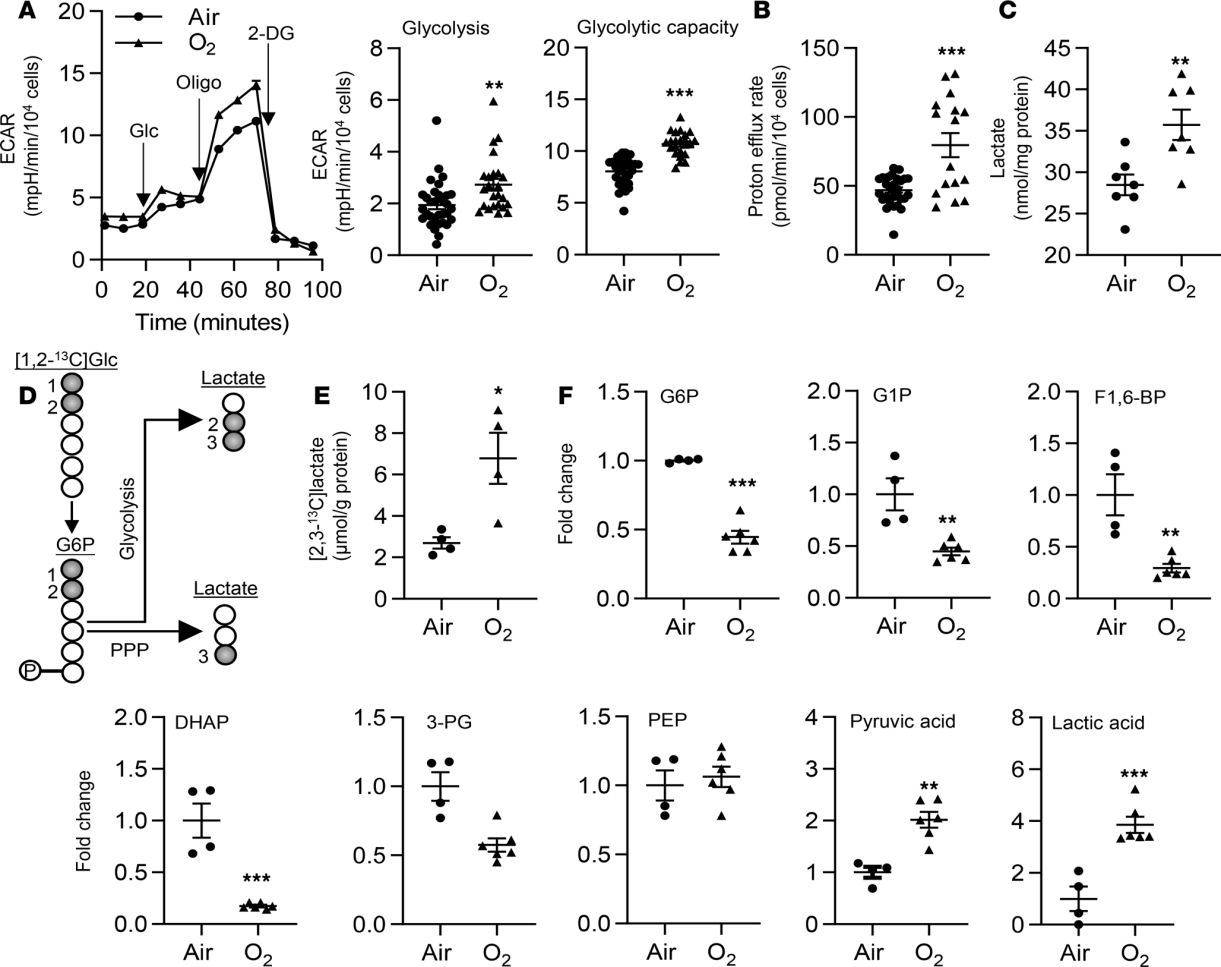
Hyperoxic exposure increases glycolysis in cultured lung ECs. (**A**–**C**, **E**, and **F**) Primary LMVECs (**A**–**C**, and **F**) and MFLM-91U cells (**E**) were exposed to hyperoxia for 24 hours and then cultured in normoxia for 24 hours (refers to O_2_). (**A**) ECAR was measured in cells by the Seahorse XF24 Analyzer. Kinetic ECAR response to glucose (Glc), oligomycin (Oligo), and 2-DG was recorded. Basal glycolysis and glycolytic capacity were calculated after normalization into the number of live cells. *n* = 32 in air and *n* = 16 in hyperoxia. (**B**) Glycolytic rate assay was performed to determine glycolysis-derived proton efflux. *n* = 32 in air and *n* = 16 in hyperoxia. (**C**) Intracellular lactate was measured using the L-lactate Assay kit. *n* = 7 per group. (**D**) Schematic figure showing the production of [2,3-^13^C]lactate and [3-^13^C]lactate from [1,2-^13^C]glucose via glycolysis and the PPP, respectively. (**E**) [2,3-^13^C]lactate was measured by the NMR when cells were incubated with [1,2-^13^C]glucose (20 mM) for 24 hours during air recovery phase. *n* = 4 per group. (**F**) Detectable intracellular metabolites during glycolysis were presented. *n* = 4 in air and *n* = 6 in hyperoxia. Data are expressed as mean ± SEM. *P < 0.05, **P < 0.01, ***P < 0.001 versus air using 1-railed *t* test (**A**–**C**, **E**, and **F**).

Although ECs rely on glycolysis for generating bioenergetics (10, 11), it is not known whether hyperoxic exposure alters glycolysis in lung ECs. Therefore, using the Seahorse Analyzer, we determined extracellular acidification (ECAR) in primary mouse lung microvascular ECs (LMVECs) exposed to hyperoxia (95% O_2_/5% CO_2_) for 24 hours, followed by air recovery (21% O_2_/5% CO_2_) for 24 hours. Exposure to hyperoxia significantly increased ECAR, which was reflected by augmented basal glycolysis and glycolytic capacity (Figure 1A). CO_2_ derived from the tricarboxylic acid cycle can contribute to the ECAR. We thus performed a glycolytic rate assay (GRA), which eliminates the contribution of CO_2_ from the tricarboxylic acid cycle to ECAR. Proton efflux rate from glycolysis was increased in lung ECs exposed to hyperoxia (Figure 1B). Furthermore, in primary mouse LMVECs, hyperoxic exposure increased the levels of intracellular lactate, as detected using an L-lactate Assay Kit (Figure 1C). Lactate can be generated from glucose through glycolysis and through the PPP (Figure 1D). Hence, we employed a metabolic flux assay to measure the levels of glycolysis-derived [2,3-^13^C]lactate by a nuclear magnetic resonance (NMR) when cells were treated with [1,2-^13^C]glucose. As shown in Figure 1E, hyperoxic exposure significantly increased levels of [2,3-^13^C]lactate in MFLM-91U cells. In addition, we performed untargeted metabolomics to detect levels of intracellular metabolites by mass spectrometry (MS) in lung ECs exposed to hyperoxia (Supplemental Figure 1; supplemental material available online with this article; https://doi.org/10.1172/jci.insight.137594DS1). Specifically, we found that the levels of glucose 6-phosphate (G6P), G1P, fructose 1,6-bisphosphate (F1,6-BP), and dihydroxyacetone phosphate (DHAP) were significantly reduced, whereas levels of glycolytic products pyruvate and lactate were increased in primary mouse LMVECs exposed to hyperoxia (Figure 1F). Protein levels of GAPDH, PFKFB3, or pyruvate kinase muscle isozyme (PKM), key enzymes in glycolysis, were not significantly altered in lung ECs exposed to hyperoxia (Supplemental Figure 2). Altogether, these data demonstrate that hyperoxic exposure significantly increases glycolytic flux in cultured lung ECs.

### Hyperoxia increases the PPP in cultured lung ECs.

**Figure 2:**
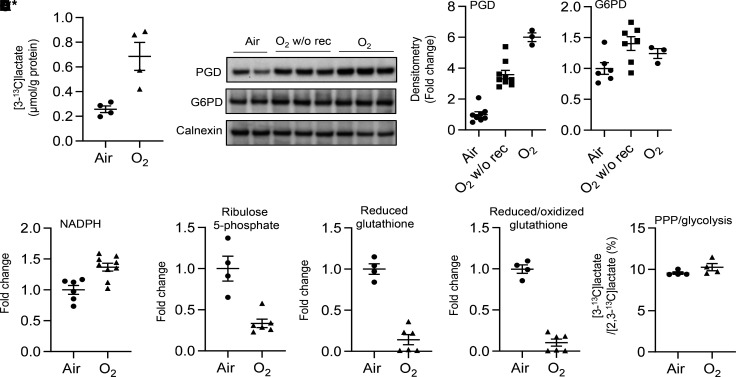
Hyperoxic exposure increases the PPP in cultured lung ECs. (**A**–**E**) MFLM-91U cells (**A** and **E**) and primary LMVECs (**B**–**D**) were exposed to hyperoxia for 24 hours and then cultured in normoxia for 24 hours (refers to O_2_) unless specifically mentioned. (**A**) [3-^13^C]lactate was measured by the NMR when cells were incubated with [1,2-^13^C]glucose (20 mM) for 24 hours during air recovery phase. *n* = 4 per group. (**B**) Western blot was performed to determine levels of PGD and G6PD proteins. O_2_ w/o rec refers hyperoxic exposure for 24 hours without air recovery, while O_2_ refers hyperoxic exposure for 24 hours, followed by air recovery for 24 hours. *n* = 6 in air, *n* = 7 in hyperoxia without air recovery, and *n* = 3 in hyperoxia with air recovery. (**C**) NADPH levels were measured using a commercially available kit. *n* = 6 in air and *n* = 9 in hyperoxia. (**D**) Levels of ribulose 5-phosphate, reduced and reduced/oxidized glutathione were determined through metabolomics analysis. *n* = 4 in air and *n* = 6 in hyperoxia. (**E**) Ratio of [3-^13^C]lactate to [2,3-^13^C]lactate was calculated based on results from Figure 1D and A****. *n* = 4 per group. Data are expressed as mean ± SEM. *P < 0.05, **P < 0.01, ***P < 0.001 versus air using 1-tailed *t* test (**A**, **C**, **D**, and **E**) or ANOVA followed by Tukey-Kramer test (**B**).

The PPP diverts G6P from glycolysis to generate NADPH and ribose 5-phosphate. We measured levels of [3-^13^C]lactate when cells were incubated with [1,2-^13^C]glucose for 24 hours, and we found that levels of [3-^13^C]lactate derived from the PPP were significantly increased in MFLM-91U cells exposed to hyperoxia (Figure 2A). Hyperoxic exposure also significantly increased protein levels of phosphogluconate dehydrogenase (PGD), the second enzyme of the oxidative arm of the PPP, as well as NADPH in primary mouse LMVECs (Figure 2, B and C). Metabolomics assays showed that hyperoxic exposure significantly decreased the levels of ribulose 5-phosphate and reduced glutathione in primary mouse LMVECs (Figure 2D). However, the ratio of [3-^13^C]lactate to [2,3-^13^C]lactate was not altered by hyperoxic exposure in MFLM-91U cells (Figure 2E). These results suggest that hyperoxic exposure increases the PPP, but does not cause a switch between the PPP and glycolysis, in lung ECs.

### Hyperoxic exposure has no effect on glucose uptake.

Glucose uptake into the cytoplasm is the first rate-limiting step of glycolysis. Whether increases in glycolysis and the PPP seen in hyperoxia are associated with augmented glucose uptake remains unclear. We thus measured the protein levels of the glucose transporters Glut1 and Glut4 by Western blot. These were not altered in MFLM-91U cells exposed to hyperoxia (Supplemental Figure 3, A and B). We also employed a 2-NBDG Glucose Uptake Assay Kit, which utilizes 2-NBDG, a fluorescently labeled deoxyglucose analog, as a probe for the detection of glucose uptake by cultured cells. As shown in Supplemental Figure 3C, hyperoxic exposure did not alter the uptake of 2-NBDG in MFLM-91U cells. In addition, mRNA and protein levels of Glut1 or Glut4 were not altered in the lungs of neonatal mice exposed to hyperoxia (Supplemental Figure 3, D and E). These data indicate that hyperoxic exposure has no effect on glucose uptake in cultured lung ECs.

### The PPP controls hyperoxia-induced proliferation in cultured lung ECs.

**Figure 3:**
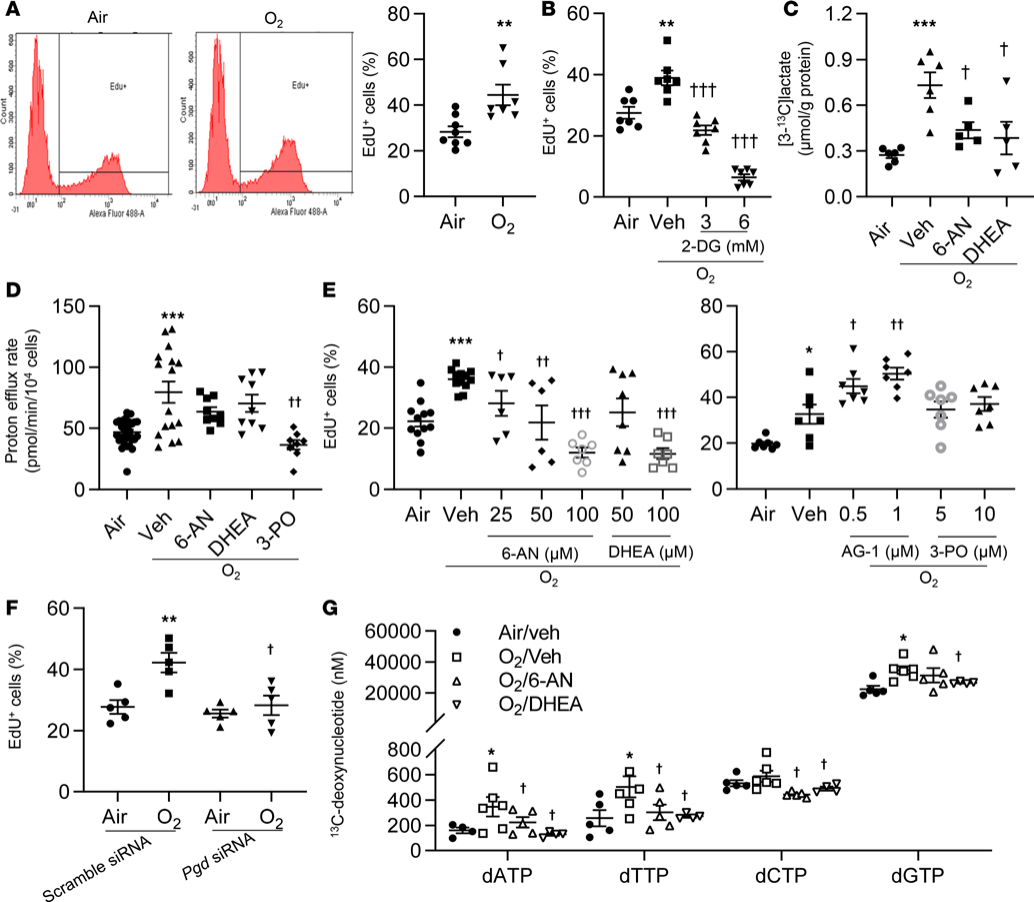
Blocking the PPP reduces hyperoxia-induced proliferation in cultured lung ECs. (**A**–**G**) Primary mouse LMVECs (**A**, **B**, **D**, **E**, **G**) or MFLM-91U cells (**C** and **F**) were exposed to hyperoxia for 24 hours, followed by normoxia for 24 hours (refers to O_2_). (**A**) EdU incorporation was measured by flow cytometry, and a percentage of EdU^+^ cells was calculated. *n* = 8 in air and *n* = 7 in hyperoxia. (**B**) EdU incorporation was measured by flow cytometry after incubation with 2-DG (3 and 6 mM, 12 hours) during air recovery phase. *n* = 7 per group. (**C**) [3-^13^C]lactate was measured by the NMR when cells were incubated with [1,2-^13^C]glucose (20 mM, 24 hours) along with 6-AN (50 μM, 12 hours) or DHEA (50 μM, 12 hours) during air recovery phase. *n* = 6 in air and O_2_/veh groups; *n* = 5 in 6-AN and DHEA treatment groups. (**D**) Glycolytic rate assay was performed after incubation with 6-AN (50 μM), DHEA (50 μM), or 3-PO (10 μM) for 12 hours during air recovery phase. *n* = 32 in air and O_2_/veh groups; *n* = 8 in 6-AN, DHEA and 3-PO treatment groups. (**E**) EdU incorporation was measured by flow cytometry after incubation with 6-AN (25–100 μM), DHEA (50 and100 μM), and 3-PO (5 and10 μM) for 12 hours during air recovery phase. *n* = 7 in per group. (**F**) EdU was measured by flow cytometry in scramble and *pgd* siRNA–transfected cells. *n* = 5 in per group. (**G**) [^13^C]-labeled deoxynucleotides were measured by mass spectrometry when cells were incubated with 20 mM [U-^13^C]glucose for 24 hours during air recovery phase. *n* = 5 per group. *P < 0.05, **P < 0.01, ***P < 0.001 versus air (**A**–**E**), scramble siRNA/air (**F**), or air/veh (**G**); ^†^*P*<0.05, ^††^*P* < 0.01, ^†††^*P* < 0.001 versus hyperoxia/vehicle (**B**–**E**, and **G**) or scramble siRNA/hyperoxia (**F**) using 1-tailed *t* test (**A**) or ANOVA followed by Tukey-Kramer test (**B**–**G**).

Although both glycolysis and the PPP can provide carbons to generate biomass, including deoxynucleotides or nucleotides, for proliferation (16), the role of glycolysis and the PPP in lung EC proliferation in response to hyperoxia is unknown. We first exposed primary mouse LMVECs to hyperoxia for 24 hours and allowed them to recover in room air for another 24 hours. Proliferation was assessed using the Click-iT EdU Cell Proliferation Kit. Hyperoxic exposure significantly increased EdU incorporation (Figure 3A). Reducing glycolysis using 2-deoxy-D-glucose (2-DG; 3 mM and 6 mM) during the air recovery phase significantly reduced hyperoxia-induced EdU incorporation (Figure 3B and Supplemental Figure 4A). 2-DG inhibits phosphoglucose isomerase and hexokinase, thereby reducing the production of G6P that can be further metabolized through glycolysis and the PPP. Thus, 2-DG could inhibit both glycolysis and the PPP. We next specifically inhibited the PPP and glycolysis using a PGD inhibitor 6-aminonicotinamide (6-AN), a glucose-6-phosphate dehydrogenase (G6PD) inhibitor dehydroepiandrosterone (DHEA), and a PFKFB3 inhibitor 3-PO. As expected, both 6-AN (50 μM) and DHEA (50 μM) incubation attenuated the hyperoxia-induced production of [3-^13^C]lactate when cells were incubated with [1,2-^13^C]glucose for 24 hours (Figure 3C), while the increase in proton efflux rate seen in hyperoxia was not affected by either 6-AN or DHEA (Figure 3D). These data suggest that both 6-AN and DHEA inhibit the PPP but not glycolysis. Functionally, both 6-AN (25–100 μM) and DHEA (50–100 μM) prevented the increase in EdU incorporation seen in hyperoxia in a dose-dependent manner (Figure 3E). Similarly, knockdown of *pgd* by siRNA transfection significantly decreased hyperoxia-induced EdU incorporation (Figure 3F and Supplemental Figure 4B). In contrast, activating the PPP, using a G6PD activator AG-1 (0.5–1 μM) (17), further increased EdU incorporation in response to hyperoxic exposure (Figure 3E). Furthermore, hyperoxic exposure significantly increased levels of [^13^C]-labeled deoxynucleotides when cells were incubated with [U-^13^C]glucose. These effects were significantly reduced by 6-AN and DHEA (50 μM) (Figure 3G). Levels of glucose-derived GTP, CTP, or UPT were not altered in any of the experimental conditions (Supplemental Figure 4C). Levels of [^13^C]-labeled ATP were not detectable when cells were incubated with [U-^13^C]glucose. Interestingly, reducing glycolysis using the PFKFB3 inhibitor 3-PO (5 μM and 10 μM) did not have any effects on hyperoxia-induced EdU incorporation (Figure 3, D and E). These results suggest that hyperoxic exposure increases the PPP, leading to increased DNA synthesis and proliferation in lung ECs.

### Inhibiting glycolysis further reduces migration in cultured lung ECs exposed to hyperoxia.

**Figure 4:**
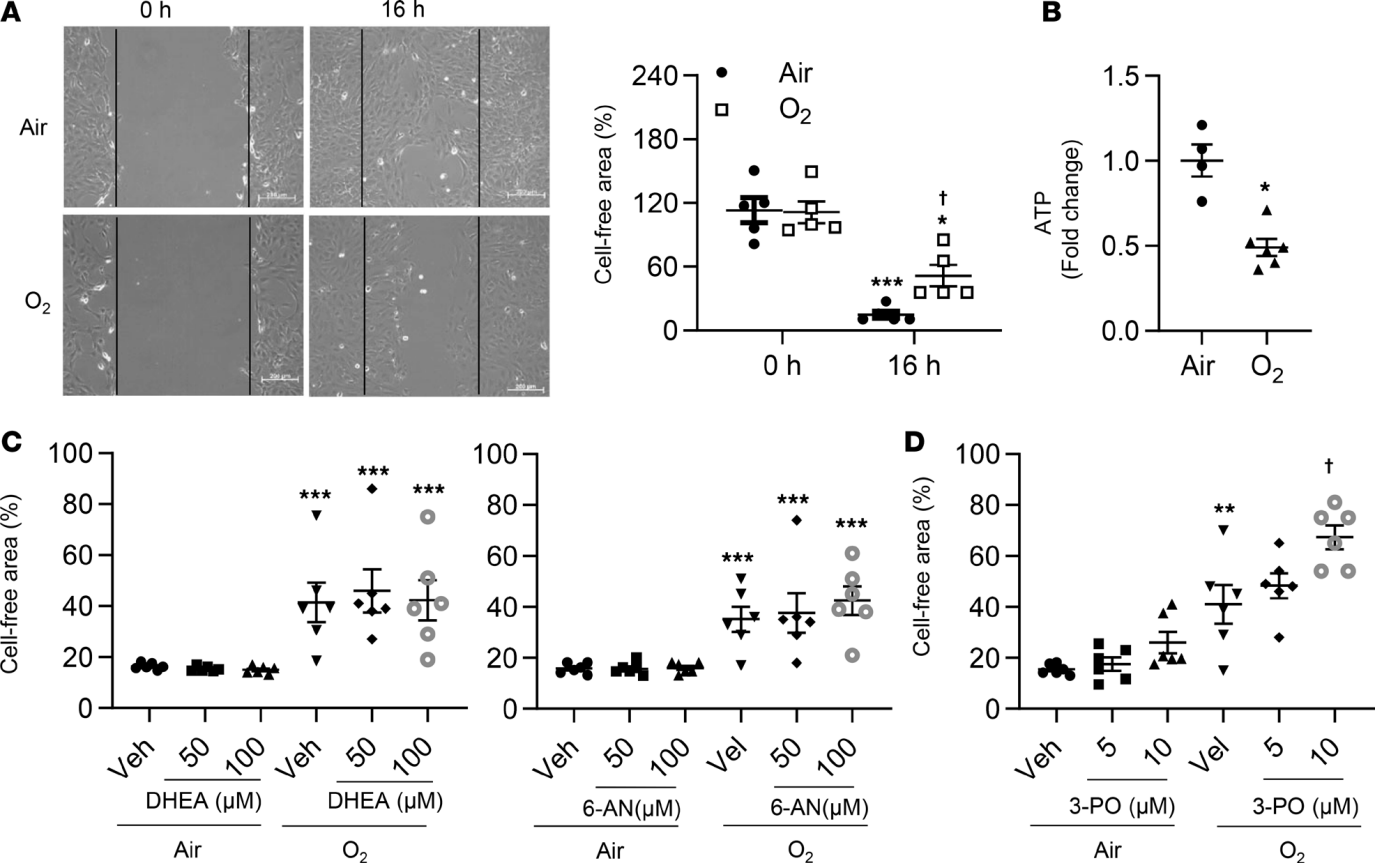
Blocking glycolysis further reduces migration in cultured lung ECs exposed to hyperoxia. Primary mouse LMVECs were exposed to hyperoxia for 24 hours, followed by normoxia for 24 hours (refers to O_2_). (**A**) Scratch assay was performed 16 hours after hyperoxic exposure, and cell-free area was calculated using ImageJ software. *n* = 5 per group. (**B**) Intracellular ATP was measured through metabolomics analysis. *n* = 4 in air and *n* = 5 in hyperoxia. (**C**) Scratch assay was performed after incubation with DHEA (50 and 100 μM), 6-AN (50 and 100 μM), or (**D**) 3-PO (5 and 10 μM) for 12 hours during air recovery phase. *n* = 6 per group. *P < 0.05, **P < 0.01, ***P < 0.001 versus air/0 hours (**A**), air (**B**)**,** or air/veh (**C** and **D**); ^†^*P* < 0.05 versus air/16 hours (**A**) or hyperoxia/vehicle (**D**) using 1-tailed *t* test (**B**) or ANOVA followed by Tukey-Kramer test (**A**, **C**, and **D**).

Hyperoxic exposure has been shown to reduce EC migration (18). It is not clear whether decreased lung EC migration persists despite air recovery. A scratch assay was performed in primary mouse LMVECs 16 hours after hyperoxic exposure to measure cell migration. As shown in Figure 4A, hyperoxic exposure followed by air recovery significantly reduced EC migration. This was associated with reduced ATP generation (Figure 4B). Incubation with DHEA (50–100 μM) or 6-AN (50–100 μM) did not affect EC migration regardless of exposure (Figure 4C). Blocking glycolysis using 3-PO (5–10 μM) further reduced migration in cells exposed to hyperoxia followed by air recovery (Figure 4D). These data indicate that glycolysis, not the PPP, modulates lung EC migration in response to hyperoxic exposure followed by air recovery.

### The PPP is increased in the lungs of mice exposed to hyperoxia as neonates and in the lungs of premature infants requiring mechanical ventilation.

**Figure 5:**
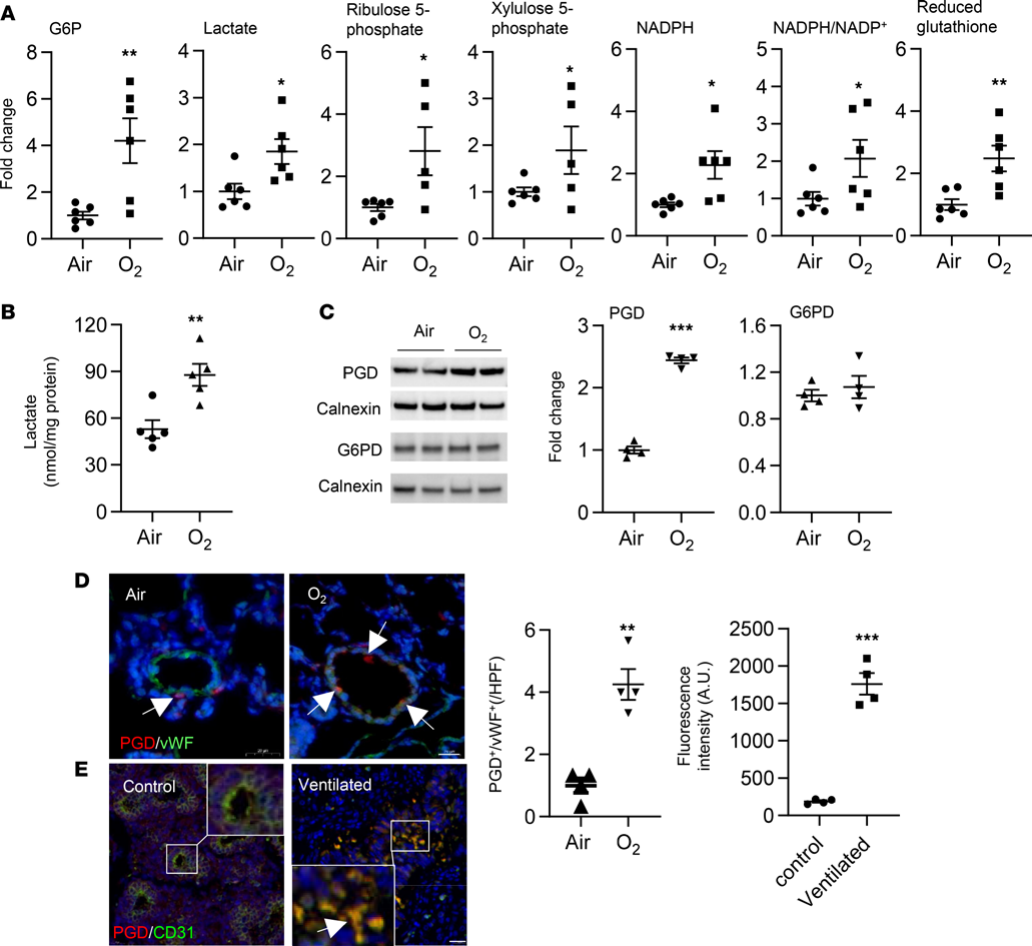
Glycolysis and the PPP are increased in lungs of mice exposed to hyperoxia, and endothelial PGD overexpression occurs in lungs of premature infants requiring mechanical ventilation. (**A**–**D**) C57BL/6J neonatal mice (<12 hours old) were exposed to air or hyperoxia (95% O_2_) for 3 days and were then allowed to recover in room air until P7 (**A**) or P14 (**B**–**D**). (**A**) Untargeted metabolomics was performed by mass spectrometry in mouse lungs, and detectable metabolites in glycolysis and the PPP were presented. *n* = 6 per group. (**B**) Lactate levels were measured in mouse lungs using a L-lactate Assay kit. *n* = 5 per group. (**C**) Western blot was performed to assess protein levels of PGD and G6PD in mouse lungs. *n* = 4 per group. (**D**) Double immunofluorescence was conducted to determine the abundance of PGD in vWF^+^ cells in mouse lungs. Numbers of PGD^+^ and vWF^+^ cells were counted in 3 randomly selected high-power fields (HPF) for each sample, which was shown in left graph. Scale bar: 20 μm. *n* = 4 per group. (**E**) Immunofluorescence was carried out to detect colocalization of PGD and CD31 in lungs of premature infants requiring mechanical ventilation. Scale bar: 20 μm. Fluorescent intensity of PGD^+^/CD31^+^ cells was evaluated using an ImageJ software, which was shown in right graph. *n* = 4 per group. Data are expressed as mean ± SEM. *P < 0.05, **P < 0.01, ***P < 0.001 versus air (**A**–**D**) or control subjects (**E**) using 1-tailed *t* test (**A**–**E**).

To determine whether our in vitro findings could be replicated in an in vivo mouse model, neonatal mice (<12 hours old) were exposed to hyperoxia for 3 days and allowed to recover in room air until P7 or P14. This model tests whether the effects of hyperoxia are persistent in vivo. Untargeted metabolomics were performed in the mouse lungs at P7 (Figure 5A and Supplemental Figure 4D). Hyperoxic exposure increased lung levels of G6P, lactate, ribulose 5-phosphate, xylulose 5-phosphate, and NADPH and reduced glutathione (Figure 5A). Similarly, the levels of lactate were increased in mouse lungs at P14 when these mice were exposed to hyperoxia as neonates (Figure 5B). Western blots showed that hyperoxic exposure increased levels of PGD protein in mouse lungs at P14 (Figure 5C). Immunofluorescence also showed that the abundance of PGD in von Wildebrand factor^+^ (vWF^+^) cells was significantly increased in mice at P14 after exposure to hyperoxia as newborns (Figure 5D). In addition, PGD expression in vWF^+^ cells was significantly increased in lungs of premature infants requiring mechanical ventilation (Figure 5E). These results suggest that the PPP is increased in the lung of hyperoxia-exposed mice and in lungs of premature infants requiring mechanical ventilation and that these effects are persistent.

### Hyperoxic exposure causes abnormal lung EC proliferation in neonatal mice.

**Figure 6:**
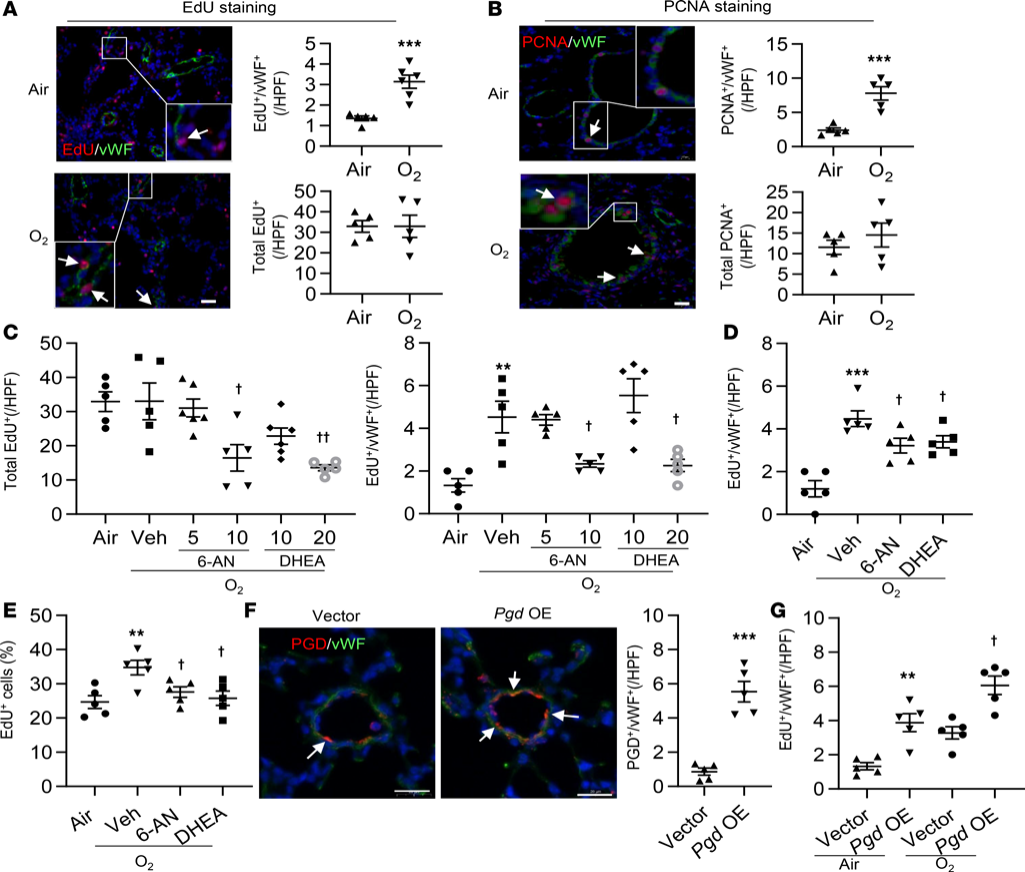
The PPP enhances lung EC proliferation in mice exposed to hyperoxia as neonates. C57BL/6J neonatal mice (<12 hours old) were exposed to air or hyperoxia (95% O_2_) for 3 days and were then allowed for recover in room air until P14. (**A**) EdU was i.p. injected at 50 mg/kg daily for 3 days before sacrificing. Lung tissues were utilized for EdU staining, along with costaining with vWF. Scale bar: 20 μm. *n* = 5 per group. (**B**) Double immunofluorescence was conducted to determine the abundance of PCNA in vWF^+^ cells in mouse lungs. Scale bar: 20 μm. *n* = 5 per group. (**C**) 6-AN (5 and 10 mg/kg, i.p.) or DHEA (10 and 20 mg/kg, i.p.) were administered daily in mice from P9 to P13. (**D**) 6-AN (10 mg/kg, i.p.) or DHEA (20 mg/kg, i.p.) were administered daily in mice from P12 to P13. (**C** and **D**) Lung tissues were utilized for double immunofluorescence of EdU incorporation and vWF. Numbers of EdU^+^ and vWF^+^ cells were counted in 3 randomly selected high-power fields (HPF) for each sample. *n* = 5 per group. (**E**) EdU incorporation was measured by flow cytometry in LMVECs isolated from hyperoxia-exposed mice treated with 6-AN (10 mg/kg) or DHEA (20 mg/kg) between P9 and P13. *n* = 5 per group. (**F**) Nanoparticles mixed with plasmid DNA expressing *pgd* or empty vector under the control of human *CDH5* promoter was administered into normoxia-exposed mice via a retro-orbital injection at P9. At P14, immunofluorescence was performed to detect colocalization of PGD and vWF in mouse lungs. *Pgd* OE, *pgd* overexpression. Scale bar: 20 μm. *n* = 5 per group. (**G**) Immunofluorescence of EdU incorporation and vWF was performed in hyperoxia-exposed mice injected with nanoparticles mixed with plasmid DNA expressing *pgd* or empty vector under the control of human *CDH5* promoter. Numbers of EdU^+^/vWF^+^ cells were counted in 3 randomly selected high-power fields (HPF) for each sample. *n* = 5 per group. Data are expressed as mean ± SEM. **P < 0.01, ***P < 0.001 versus air (**A**–**E**), vector (**F**), or air/vector (**G**); ^†^*P* < 0.05, ^††^*P* < 0.01 versus hyperoxia/vehicle (**C**–**E**) or hyperoxia/vector (**G**) using 1-tailed *t* test (**A**, **B**, and **F**) or ANOVA followed by Tukey-Kramer test (**C**–**E**, and **G**).

To determine whether neonatal hyperoxic exposure affects EC proliferation in mouse lungs at P14, we performed EdU staining using the Click-it EdU Assay kit. Although there were no changes in the total number of lung EdU^+^ cells between air and hyperoxia groups, hyperoxic exposure significantly increased the number of EdU^+^/vWF^+^ lung EC cells (Figure 6A). Similarly, the expression of PCNA in vWF^+^ cells was significantly increased by hyperoxic exposure (Figure 6B). Altogether, these results demonstrate that hyperoxic exposure causes abnormal lung EC proliferation in mouse lungs beyond the neonatal period.

### The PPP contributes to hyperoxia-induced abnormal lung EC proliferation and dysmorphic angiogenesis.

To determine the role of the PPP in modulating lung EC proliferation, mice were injected with DHEA (5–10 mg/kg, i.p.) or 6-AN (10–20 mg/kg, i.p.) daily for 5 days between P9 and P13, or for 2 days between P12 and P13. The total number of EdU^+^ cells was significantly reduced with DHEA or 6-AN treatment at P14 in mice exposed to hyperoxia as newborns, and this occurred in a dose-dependent manner (Figure 6C). Lung EC proliferation, demonstrated by colocalization of EdU and vWF, was also significantly reduced at P14 by either DHEA or 6-AN administration in a dose- and time-dependent manner (Figure 6, C and D). Similarly, EdU incorporation was reduced in primary mouse LMVECs isolated from hyperoxia-exposed mice treated with DHEA (10 mg/kg, i.p.) or 6-AN (20 mg/kg, i.p.) daily for 5 days compared with vehicle controls (Figure 6E). In contrast, nanoparticle-mediated endothelial *pgd* overexpression further increased hyperoxia-induced EC proliferation in mouse lungs (Figure 6, F and G). These data demonstrate that inhibiting the PPP prevents, while endothelial *pgd* overexpression further increases, hyperoxia-induced abnormal lung EC proliferation.

We next measured lung levels of CD31, another EC marker, and found that hyperoxic exposure did not alter CD31 protein in mouse lungs, nor were lung CD31 protein levels changed by DHEA (20 mg/kg) or 6-AN (10 mg/kg) (Supplemental Figure 5A). However, increased CD31 abundance was observed within the lung interstitium of mice exposed to hyperoxia as neonates and recovered in room air until P14. The CD31 signal was also not adjacent to distal airways. Furthermore, CD31 IHC indicates that neonatal hyperoxic exposure caused vessel occlusion in mouse lungs (Supplemental Figure 5B). These lung abnormalities resulting from hyperoxic exposure in the neonatal period were attenuated by DHEA and 6-AN (Supplemental Figure 5B). These data indicate that hyperoxic exposure increases abnormal lung EC proliferation, leading to persistent dysmorphic angiogenesis, which is attenuated by blockade of the PPP.

### Endothelial pgd overexpression augments, while inhibiting the PPP protects against, hyperoxia-induced alveolar simplification in neonatal mice.

**Figure 7:**
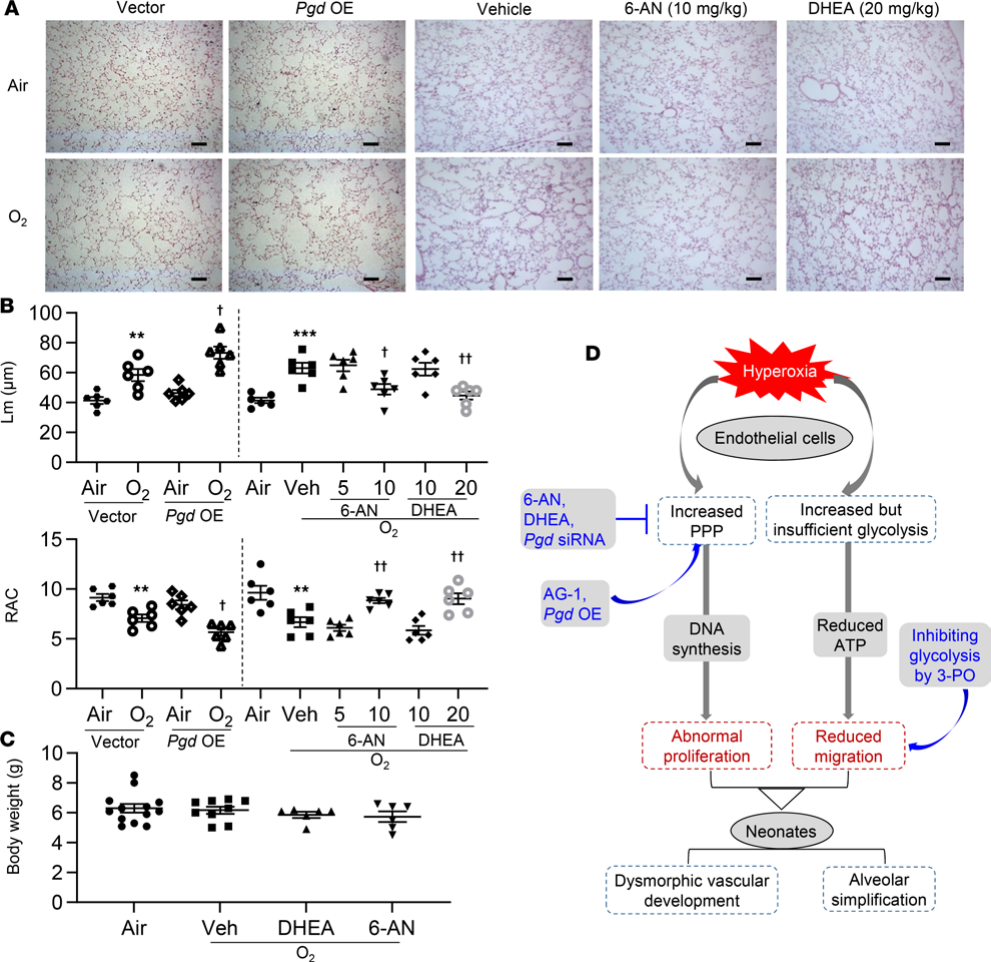
Endothelial *pgd* overexpression augments, whereas blocking the PPP attenuates, alveolar simplification in neonatal mice exposed to hyperoxia. C57BL/6J neonatal mice (<12 hours old) were exposed to air or hyperoxia (95% O_2_) for 3 days and were then allowed for recover in room air until P14. At P9, mixtures of nanoparticles and plasmid DNA expressing *pgd* or empty vector under the control of human *CDH5* promoter were administered into mice via a retro-orbital injection. 6-AN (5 and 10 mg/kg, i.p.) or DHEA (10 and 20 mg/kg, i.p.) were administered daily in mice from P9 to P13. (**A**) H&E staining was performed to assess lung morphology in mouse lungs. *Pgd* OE, *pgd* overexpression. Scale bar: 100 μm. (**B**) Mean linear intercept (Lm) and radical alveolar count (RAC) were calculated in mouse lungs. *n* = 6 per group. (**C**) Body weight was calculated after 6-AN or DHEA administration in neonatal mice exposed to hyperoxia. *n* = 6 per group. (**D**) Schematic showing that hyperoxic exposure increased the PPP and glycolysis in lung ECs. Hyperoxia-induced increase in the PPP results in abnormal EC proliferation and subsequent dysmorphic vascular development and alveolar simplification in neonates. Data are expressed as mean ± SEM. **P < 0.01, ***P < 0.001 versus air/vector or air; ^†^*P* < 0.05, ^††^*P* < 0.01 versus hyperoxia/vector or hyperoxia/vehicle using ANOVA followed by Tukey-Kramer test (**A**–**C**).

Neonatal hyperoxic exposure has been shown to cause alveolar simplification despite air recovery (19). Thus, we determined the effect of the PPP on hyperoxia-induced alveolar simplification, as reflected by increased mean linear intercept (Lm) and reduced radial alveolar count (RAC). As expected, hyperoxic exposure increased Lm and reduced RAC in the lungs of neonatal mice at P14 (Figure 7, A and B). These effects were further augmented in mice injected with nanoparticles mixed with plasmid DNA expressing *pgd* under the control of the human *CDH5* promoter (Figure 7, A and B). Although treatment with both DHEA (20 mg/kg, i.p.) and 6-AN (10 mg/kg, i.p.) for 2 days did not influence hyperoxic lung injury (Supplemental Figure 5C), treatment for 5 days protected hyperoxia-exposed mice, against an increase in Lm and reduction in RAC (Figure 7, A and B). Neither DHEA (20 mg/kg) nor 6-AN (10 mg/kg) affected body weight, regardless of the exposure (Figure 7C). Altogether, the data demonstrate that increased PPP contributes to hyperoxia-induced alveolar simplification in mouse lungs.

## Discussion

Here, we show that hyperoxic exposure increased both glycolysis and the PPP in cultured lung ECs and in neonatal mouse lungs. Mouse lungs at birth are structurally similar to human neonates born at 30–34 weeks of gestation. Hyperoxic exposure in neonatal mice can be used to mimic lung injury in BPD patients. Our model allows hyperoxia-exposed mice to recover in normoxia so as to test whether the impact of hyperoxia is persistent. Hyperoxic exposure in neonatal mice followed by air recovery caused abnormal lung EC proliferation and dysmorphic vascular development. Blocking the PPP protected against neonatal hyperoxia–induced abnormal lung EC proliferation and alveolar simplification in mice. Therefore, the PPP controls dysmorphic vascular growth during neonatal hyperoxic lung injury and recovery.

The PPP serves to generate NADPH and nucleotides for cell proliferation and growth (20). We show that hyperoxic exposure increased NADPH levels in both cultured lung ECs and mouse lungs. This may be used for generating or replenishing reduced glutathione, as oxidized glutathione was increased in hyperoxia-exposed cultured lung ECs and mouse lungs. The sugar backbones of nucleotides are derived from ribose 5-phosphate during the PPP. Surprisingly, ribulose 5-phosphate was reduced in cultured lung ECs exposed to hyperoxia, possibly due to increased consumption of ribulose 5-phosphate for (deoxy)nucleotide synthesis. In fact, hyperoxic exposure increased glucose-derived deoxynucleotide synthesis and proliferation in cultured lung ECs, which was attenuated by pharmacological inhibition of the PPP. In the lungs of mice exposed to hyperoxia as neonates, levels of ribulose 5-phosphate and xylulose 5-phosphate were increased. This may be due to a persistent anabolic state and/or increased PPP in other lung cells. This is corroborated by the findings that hyperoxia stimulates the expansion of alveolar epithelial type II cells in neonatal mice (21). Altogether, hyperoxic exposure augments the PPP, leading to increased DNA synthesis and proliferation in lung ECs. These ECs are differentiated into stalk cells for proliferation during sprouting angiogenesis. Whether hyperoxic exposure causes differentiation of ECs into stalk cells for proliferation remains to be investigated. Although endothelial PGD was increased in the lungs of premature infants requiring mechanical ventilation, it remains elusive whether other risk factors for BPD, including prematurity, infection, and genetic predisposition, affect endothelial PPP.

In CHO cells, hyperoxic exposure significantly increased glycolysis in order to generate bioenergetic substrates in an environment of impaired mitochondrial respiration (22). This is in agreement with our previous and present findings that hyperoxic exposure followed by air recovery decreases oxidative phosphorylation but increases glycolysis in lung ECs (19). In contrast to our findings, hyperoxic exposure reduced glycolytic capacity and glycolytic reserve in mouse lung epithelial cells (MLE-12), and it decreased lactate release in 3T3-L1 adipocytes (14, 23). Furthermore, hyperoxic exposure reduced glucose uptake in 3T3-L1 adipocytes (23), decreased Glut1 and Glut4 proteins in skeletal muscle from the hind limb (24), and lowered Glut4 expression in adult rat lungs (25). In our study, the glucose transporters Glut1 and Glut4 were not altered, nor was glucose uptake in lung ECs or neonatal mouse lungs exposed to hyperoxia. The discrepancies in how hyperoxia modifies glycolysis and glucose uptake may be cell specific. Currently, we are investigating whether glycolysis and the PPP are jointly upregulated via an overarching set of master regulators or separately modulated in parallel in response to hyperoxic exposure.

ECs rely mainly on glycolysis instead of oxidative metabolism for ATP production, of which 40% is utilized for the proliferation and migration (10). Although glycolysis was increased by hyperoxic exposure, intracellular ATP in cultured lung ECs was significantly reduced. This suggests that increased glycolysis may be a compensatory mechanism for generating ATP after hyperoxic exposure. Endothelial tip cells are responsible for migration via filopodia extension, a process requiring ATP as an energy source (10). Reduced intracellular ATP may explain why hyperoxic exposure reduced migration of cultured lung ECs. Selective inhibition of glycolysis further reduced migration of lung ECs, suggesting that glycolysis-derived ATP plays an important role in promoting EC migration. In contrast, inhibiting glycolysis did not affect proliferation of lung ECs. This indicates that the PPP and glycolysis differentially control proliferation and migration in lung ECs. This is in agreement with the findings that, in glioblastoma cells, proliferation and migration are differentially modulated by the PPP and glycolysis (26). Further studies are required to determine whether the PPP and glycolysis specifically modulate stalk and tip cells, which are responsible for proliferation and migration, respectively, in response to hyperoxia exposure. It is interesting to note that lung ATP levels were significantly increased in the mice exposed to hyperoxia as neonates. This may be due to increased ATP release and breakdown into the extracellular space by hyperoxia (27). Increased extracellular ATP may also mediate activation of the hyperoxia-induced inflammasome or provide signals for cell survival (28, 29).

Impaired pulmonary angiogenesis appears to be key in the pathogenesis of BPD (30) as inhibiting angiogenesis worsens, whereas VEGF145 gene delivery attenuates hyperoxia-induced pulmonary alveolar simplification in neonatal rats (4, 6). In premature infants with BPD, increased pulmonary vascular growth is observed; however, the vessels formed are dysmorphic (8, 9). These abnormal capillaries frequently locate within thickened alveolar septa but are not immediately adjacent to the alveolar epithelium, and they are characterized by decreased vascular branching, as well as a dual capillary pattern (8, 9). In addition, total EC proliferation and volume are augmented in ventilated infants, which is associated with increased parenchymal volume (8). In the present study, abnormal lung EC proliferation and dysmorphic angiogenesis were also observed in mice at P14 when these mice were exposed to hyperoxia as newborns. This would result in reduced efficiency of gas exchange, despite the expansion of the pulmonary microvasculature during hyperoxic lung injury. By pharmacologically inhibiting the PPP, we were able to ameliorate the hyperoxia-induced abnormal lung EC proliferation, dysmorphic lung vasculature, and alveolar simplification. It is important to note that, although treatment with PPP inhibitors for a shorter duration (2 days) reduced lung EC proliferation, this did not protect against hyperoxic lung injury. Specifically increasing endothelial proliferation via the PPP by nanoparticle-mediated *pgd* overexpression aggravated hyperoxic lung injury. These findings suggest that abnormal EC proliferation contributes to hyperoxia-induced dysmorphic angiogenesis and that reduced lung EC proliferation by inhibiting the PPP is not the result from the improvement of lung injury. Use of EC-specific G6PD- or PGD-KO mice could further validate the role of the PPP in neonatal hyperoxic lung injury.

As an end-product of glycolysis, lactate plays an important role in energy generation, immune tolerance, and angiogenesis (31–33). Lactate in fact diffuses further away from vessels than oxygen (34). Perhaps lactate diffusion into the relatively hypoxic interstitium leads to proliferation and dysmorphic angiogenesis of ECs ectopically located there. Hyperoxic exposure increased CD31 protein levels in female human lung ECs isolated from donors at 18–24 weeks gestational age, and in adult mouse lungs (35, 36). We did not observe changes in lung CD31 protein levels in mice exposed to hyperoxia as neonates. We also did not explore sex differences in lung CD31 in our model. The ontogeny of lung CD31 could not be clarified in our study because we only evaluated postnatal samples.

It has been shown that 30% of infants with moderate to severe BPD develop pulmonary hypertension (37, 38). In murine models, neonatal hyperoxic exposure causes pulmonary vascular remodeling and right ventricular hypertrophy, characteristics of pulmonary hypertension (39, 40). We also reported that, in mice, hyperoxic exposure for 3 days as neonates causes pulmonary vascular remodeling, vessel obliteration, and pulmonary hypertension in adulthood (41). Ongoing studies will further determine whether neonatal hyperoxia chronically increases the PPP and EC proliferation, leading to vessel obliteration and subsequent pulmonary hypertension in adulthood.

In conclusion, neonatal hyperoxic exposure increases glycolysis and the PPP, which control lung EC migration and proliferation, respectively. Endothelial *pgd* overexpression aggravates, whereas inhibiting the PPP protects against, hyperoxia-induced dysmorphic angiogenesis and subsequent alveolar simplification in mouse lungs (Figure 7D). Our findings provide metabolic mechanisms and potential therapeutic approaches targeting the PPP to prevent dysmorphic vascular development and the development of BPD in premature infants.

## Methods

### Cell culture.

Mouse fetal lung EC lines (MFLM-91U cells) were purchased from Seven Hills Bioreagents and cultured in UltraCulture medium (BioWhittaker) (19). There was no mycoplasma contamination in these cells. Primary mouse LMVECs were isolated from neonatal mice (3–5 days old) and characterized as we previously described (19). In a separate experiment, LMVECs were isolated from hyperoxia-exposed mice treated with 6-AN and DHEA for in vitro EdU incorporation assays. These cells were cultured in dishes/plates precoated with 30 μg/mL human fibronectin using VascuLife EnGS-Mv medium, which was changed every 24 hours. Primary LMVECs at passages 4–5 were used for experiments. Cells were incubated with 2-DG (3 mM and 6 mM), DHEA (50 μM and 100 μM), 6-AN (25 μM, 50 μM, and 100 μM), or AG-1 (0.5 μM and 1 μM) for 12 hours during the air recovery phase. MFLM-91U cells were transfected with scramble or *pgd* siRNA (10 nM) for 48 hours with lipofectamine 3000 (Invitrogen) (Table 1).

### Hyperoxic exposure.

Cells at 70%–80% confluence were exposed to hyperoxia (95% O_2_ and 5% CO_2_) or air (21% O_2_ and 5% CO_2_) for 24 hours, followed by normoxia for 24 hours (19). Culture media was changed every 24 hours. Newborn C57BL/6J mice (<12 hours old, male and female) along with their mothers were exposed to room air or hyperoxia (>95% O_2_) for 72 hours in an A-chamber (BioSpherix) (19). The dams were switched every 24 hours between room air and hyperoxia to avoid injury. The pups were allowed to recover in air until P7 or P14.

### Administration of DHEA or 6-AN in mice.

Lung alveolar formation starts at P4, and it peaks between P10 and P14 (42, 43). Thus, DHEA (10 and 20 mg/kg) (44) or 6-AN (5 and 10 mg/kg) (45) was administered daily through i.p. injection to mice for 5 days (between P9 and P13) after they were exposed to hyperoxia for 3 days as neonates. In a separate experiment, we injected hyperoxia-exposed mice with DHEA (20 mg/kg, i.p.) or 6-AN (10 mg/kg, i.p.) for 2 days (between P12 and P13). At P14, mice were sacrificed and lungs were used for further analysis.

### Nanoparticle-mediated endothelial pgd overexpression.

The EndoNP1 nanoparticles were provided by MountView Therapeutics LLC. The nanoparticles were mixed with plasmid DNA expressing *pgd* or empty vector under the control of human *CDH5* promoter at an optimized ratio of 1 μg plasmid DNA to 3 μL nanoparticles for 10 minutes at room temperature. Each C57BL/6J mouse received 3 μg of plasmid DNA via a retro-orbital injection at P9 (46). Mice were sacrificed at P14.

### Lung tissues from premature infants.

Human lung samples were obtained from premature infants between 23 and 29 weeks postmenstrual age, who lived 5–15 days and required mechanical ventilation, and controls were premature infants who were not mechanically ventilated and survived less than 24 hours, as described previously (8).

### Measurement of glycolysis.

ECAR was measured using a Seahorse XF24 Analyzer (Seahorse Bioscience; ref. 47). In brief, cells at 20,000 cells/well were seeded in Seahorse XF24 microplates overnight before ECAR measurement. Cells were incubated with XF base medium supplemented with 1 mM glutamine for 45 minutes to 1 hour in a non-CO_2_ incubator at 37°C. Meanwhile, a prehydrated Seahorse XF Sensor Cartridge was loaded with glucose (100 mM; MilliporeSigma), oligomycin (100 μM; MilliporeSigma), and 2-DG (500 mM; MilliporeSigma) into ports A, B, and C, respectively. The Seahorse XF Analyzer was calibrated, and the assay was performed using the Seahorse XF Glycolysis Stress Test Assay protocol as suggested by the manufacturer (47). Numbers of living cells in representative wells were counted before the Seahorse assay, and these cell counts were used to normalize ECAR.

### GRA analysis.

Cells were incubated with the XF base medium without phenol red, which was supplemented with 5.0 mM HEPES (Thermo Fisher Scientific), 10 mM glucose, 2 mM glutamine (Caisson Labs), and 1 mM pyruvate (Invitrogen) for 1 hour in a non-CO_2_ incubator at 37°C. Meanwhile, a prehydrated Seahorse XF Sensor Cartridge was loaded with rotenone/antimycin A (50 μM) and 2-DG (500 mM) into port A and port B, respectively. The Seahorse XF Analyzer was calibrated, and the assay was performed using the Seahorse XF GRA protocol as suggested by the manufacturer. Proton efflux rate was recorded, which was normalized to numbers of living cells in representative wells.

### Metabolomics assay.

At least 1 million cells were collected by adding 2 mL of chilled methanol (MX0486-1, MilliporeSigma), or 50 mg of lung tissues were homogenized with 2 mL of chilled methanol. These mixtures were combined with 4 mL of HPLC grade chloroform (A452-1, MilliporeSigma) on ice. The mixtures were vortexed for 1 minute, incubated in an ultrasound bath for 5 minutes, and then combined with 2 mL of HPLC grade water (WX0001-1, MilliporeSigma). The samples were vortexed and centrifuged at 1800*g* for 10 minutes at 4°C. The top layer (aqueous phase) was collected for untargeted metabolomics analysis using a Thermo Fisher Scientific Ultimate 3000 LC coupled with a Q-Exactive Plus mass spectrometer. A total of 5 μL of each sample was injected on a Zic-pHILIC Column (150 × 2.1 mm, 5 μm particles, EMD Millipore). The mobile phases were (A) 20 mM ammonium carbonate in 0.1 % ammonium hydroxide and (B) acetonitrile 97% in water. The gradient conditions were as follows: 100% B at 0 minutes, 40% B at 20 minutes, 0% B at 30 minutes for 5 minutes, and then back to 100% B in 5 minutes, followed by 10 minutes of reequilibration. Full MS spectra were acquired in switching polarity at 70,000 resolution, covering a range of mz 66 to 1000. Compound discoverer 3.0 (CD, Thermo Fisher Scientific) was used to generate a list of features (mass/charge ratio [mz] and retention time) found in a pool sample (pool of all samples). This list was used as an inclusion list for MS/MS runs in positive and negative ion modes separately. Using CD, the features that were selected for MS/MS in the first MS/MS run were then removed from the inclusion list, and the MS/MS experiments were repeated with the new list. By repeating this process 4 times, we were able to obtain MS/MS data for most of the features detected by CD. All the data were then combined and analyzed in CD. Likely elemental compositions were computed based on the accurate mass and isotope pattern, and a mzCloud MS/MS spectra database, a local MzVault database, and chemspider libraries were searched to identify possible candidates (48). Each compound was then manually curated to ensure proper integration, and the accuracy of the mzCloud/mzVault hits were also checked. Compounds with a high-quality MS/MS library match were assigned the name of their match.

### Measurement of glucose-derived deoxynucleotides or nucleotides by MS.

One million to 2 million cells were cultured in a 60 mm dish with DMEM medium containing 20 mM [U-^13^C]glucose (Cambridge Isotope Laboratories) for 24 hours during the air recovery phase after hyperoxic exposure. Intracellular [^13^C]-labeled deoxynucleotides or nucleotides from [U-^13^C]glucose were detected using the MS as described previously (49). Briefly, cells were washed with 2 mL ice-cold 150 mM ammonium acetate (NH_4_AcO, pH 7.3). A total of 1 mL of –80°C cold 80% methanol was added, which was incubated at –80°C for 20 minutes. Cells were then scraped off, and supernatants were transferred into microfuge tubes. Samples were pelleted at 4°C for 5 minutes at 14,000 rpm, and the supernatants were transferred into an Eppendorf microfuge tube. Cell pellets were reextracted with 200 μL ice-cold 80% MeOH and spun down, and the supernatants were combined. Samples were dried at room temperature under a vacuum and resuspended in 100 μL of acetonitrile containing 10 μM of adenosine-^13^C10,^15^N5-monophosphate (labeled AMP, Sigma Aldrich) as internal standard (IS). A 6-point standard curve was prepared in the same acetonitrile plus IS from pure chemical standards (ATP, CTP, GTP, UTP, dATP, dGTP, dCTP, and dTTP; all from VWR) as a 1- to 10-dilution series with 100 μM as the highest concentration. Samples were analyzed by liquid chromatography–MS (LC-MS) on a Vanquish LC coupled to an ID-X MS (Thermo Fisher Scientific). Sample or standard (5 μL) was injected on a ZIC-pHILIC peek-coated column (150 mm × 2.1 mm, 5 μm particles, maintained at 40°C; Sigma-Aldrich). Buffer A was 20 mM ammonium carbonate, 0.1% ammonium hydroxide in water; Buffer B was 97% acetonitrile in water. The LC program was as follows: starting at 99% B, to 40% B in 17 minutes, then to 0% B in 10 minutes, maintained at 0% B for 5 minutes, then back to 99% B in 4 minutes, and reequilibrated at 99% B for 11 minutes. The flow rate was maintained at 0.15 mL/min. Data were acquired on the ID-X in switching polarities at 500,000 resolution, with an AGC target of 1 × 10^5^, and a *m/z* range of 65 to 1000. The combined extracted ion chromatogram for all *m/z* corresponding to all isotopologues for each compound was plotted, and the corresponding peak area was integrated. The total combined concentration of all isotopologues of each compound was calculated using this area divided by the area of the IS and using the standard curves. The relative intensity of each isotopologue for each compound was obtained by combining the mass spectra of the 3 scans at the top of each peak. In the obtained mass spectrum, the intensity of each isotopologue’s *m/z* was extracted and used to calculate the relative abundances.

### Measurement of glucose-derived lactate by the NMR.

Cells were incubated with 20 mM [1,2-^13^C]glucose (Cambridge Isotope Laboratories) for 24 hours during the air recovery phase after hyperoxic exposure. The extraction of intracellular metabolites and the NMR analysis of [^13^C]-labeled lactate from [1,2-^13^C]glucose were performed as previously described (50). In brief, ^1^H-NMR Spectroscopy High-resolution ^1^H-NMR spectra of extracellular metabolites were obtained on a Bruker 600 spectrometer operating at 600 MHz after normalizing the samples by DSS (Thermo Fisher Scientific). ^1^H-NMR spectra of extracellular extracts were acquired using a 9.6 kHz spectral width and 32 K data points. The acquisition time was 1.7 seconds, and the relaxation delay was 10 seconds with 128 scans. Proton decoupled ^13^C spectra were acquired on a Bruker 600 spectrometer. Proton decoupling was performed using a Waltz-16 sequence. ^13^C NMR parameters include a relaxation delay of 30 seconds, an acquisition time of 1 second, and spectral width of 36 kHz. Further quantitative processing of the experimental results was performed with proprietary software implemented in the software package MestReNova.

### Measurement of glucose uptake.

Glucose uptake was performed by detecting the uptake of 2-NBDG by culture cells using the 2-NBDG Glucose Uptake Assay Kit (Abcam) according to the manufacturer’s instructions. In brief, cells were incubated with glucose-free HBSS medium for 30 minutes after washing with PBS. After washing with PBS, cells were incubated with 2-NBDG (100 μM) for 30 minutes at 37°C. Cells were then resuspended in 150 μL of prechilled PBS containing 0.5% BSA, and flow cytometry analysis was performed within 30 minutes.

### Measurement of lactate levels.

L-lactate was measured in cells and lung tissues using the L-lactate Assay kit (ab65331, Abcam) according to the manufacturer’s instructions. All samples were deproteinized with the Deproteinizing Sample Preparation Kit-TCA (ab204708, Abcam) before analysis. Protein concentration in samples before deproteinization was measured for normalization.

### Determination of NADPH levels.

Levels of NADPH were determined in cells and lung tissues using the kit from Abcam (ab65349, Abcam) according to the manufacturer’s protocol. Briefly, 50 mg of tissue or 2 × 10^6^ cells were pelleted and lysed in 800 μL extraction buffer provided using a Dounce homogenizer. DNA was sheared with a needle in lysates, and the enzymes consuming NAHPD were removed by filtering the samples through a 10 kD spin column (ab93349, Abcam). A total of 200 μL of lysates was heated at 60°C for 30 minutes to decompose NADP^+^ while NADPH was intact, which was used for assessing NADPH concentration. Protein concentration in samples before deproteinization was measured for normalization.

### Evaluation of cell proliferation.

In vitro proliferation was determined by Click-iT EdU Flow Cytometry Assay Kit from Invitrogen according to the manufacturer’s protocol. Briefly, cells were incubated with 10 μM of a nucleoside analog of thymidine (5-ethynyl-2′deoxyuridine, EdU) for 2 hours. After washing and fixation, incorporated EdU during DNA synthesis was labeled with Alexa Fluor 488 azide (Thermo Fisher Scientific) in the provided reaction buffer for 30 minutes. Cells were analyzed using the BD FACSAria (BD Bioscience).

For mouse lung EdU detection, mice will be i.p. injected with EdU (50 mg/kg, daily) for 3 days and then sacrificed at 24 hours after the last EdU injection. Click-iT EdU Cell Proliferation Kit for Imaging, Alexa Fluor 647 was used to determine proliferation in lungs, which were costained with the EC marker vWF as described previously (51). The numbers of EdU^+^ cells were counted in 3 randomly selected high-power fields (HPF) using a Zeiss Axiovert 200M Fluorescence Microscope.

### Determination of cell migration.

Cell migration was assessed in cells using the Scratch assay (52). Once reaching approximately 90% confluence, the bottom of the culture plates was scratched using a 200 μL pipette tip. Cells were then washed with PBS and cultured in medium containing 0.1% FBS, mitomycin C (20 μm), and VEGF (50 ng/mL) for 16 hours. Images were captured at ×100 magnification on a Zeiss Axiovert 200M Microscope. Cell-free area was assessed to negatively reflect migration using MiToBo analyzer software in ImageJ (NIH) (52).

### Determination of protein levels.

Western blot was performed as we described previously (19). The membranes were blocked for 1 hour at room temperature with 5% BSA and then probed with 1:200–1:1000 diluted antibodies against PGD, G6PD, PKM, PFKFB3, Glut1, Glut4, and GAPDH to determine the corresponding proteins. Information of antibodies is shown in Table 1. Protein levels were detected using secondary antibodies (1: 5,000 dilutions in 5% BSA in PBS containing 0.1% Tween [v/v] 20 for 1 hour) linked to horseradish peroxidase (Vector Laboratories), and bound complexes were detected by the ChemiDoc Touch Imaging System (Bio-Rad) using the enhanced chemiluminescence method (MilliporeSigma). Equal loading of the samples was determined by quantification of proteins, as well as by reprobing membranes for the housekeeping control calnexin.

### Determining steady-state mRNA levels.

Total RNA was extracted by the TRIzol reagent and purified using the RNeasy miniprep kit (Qiagen). RNA samples were quantified by the NanoDrop One Microvolume UV-Vis Spectrophotometer (Thermo Fisher Scientific). Then, 400 ng of total RNA were used for reverse transcription using the Taqman Reverse Transcription Reagents (Thermo Fisher Scientific). A total of 1 μL of cDNA was used for real-time PCR reactions by the 7300 Real-Time PCR System (Applied Biosystems). All Taqman gene probes were purchased from Thermo Fisher Scientific (Table 1). Gene expression was normalized to 18s rRNA levels. Relative RNA abundance was quantified by the comparative 2^–ΔΔCt^ method.

### Immunofluorescence.

Lung sections were deparaffinized and subjected to heat-mediated antigen retrieval in a citrate buffer solution (Vector Labs) and then stained overnight at 4°C with antibodies against PGD, PCNA, vWF, and CD31 (Table 1). After incubation with secondary antibodies for 1 hour at room temperature, sections were mounted in hard-set mounting medium containing DAPI (Vector Labs) and allowed to incubate overnight. Colocalization of PGD^+^/vWF^+^ and PCNA^+^/vWF^+^ cells in mouse lungs were counted in 3 randomly selected HPF using a Zeiss Axiovert 200M Fluorescence Microscope. Fluorescent intensity of PGD^+^/CD31^+^ cells in human lungs was evaluated using an ImageJ software. These experiments were carried out in a blinded manner.

### Evaluating lung morphometry.

Lm and RAC were measured in mouse lungs stained with H&E as previously described (19). In brief, we inflated nonlavaged mouse lungs with 1% low-melt agarose at a pressure of 25 cm H_2_O, and we fixed them with 4% neutral buffered paraformaldehyde. These fixed lungs were embedded in paraffin and sectioned into 4 μm sections using a rotary microtome (MICROM International GmbH). Lung midsagittal sections with H&E staining were utilized to determine airspace Lm. A perpendicular line was drawn from the center of the respiratory bronchiole to the distal acinus (as defined by the pleura or the nearest connective tissue septum). The number of septa intersected by each line was counted as RAC, and a minimum of 8 counts were performed for each animal.

### Statistics.

Statistical analyses were performed using GraphPad Prism 8. The results were expressed as mean ± SEM. The 1-tailed *t* test was used for detecting statistical significance of the differences between means of 2 groups after checking the normality of data. The statistical significance of the differences among groups was evaluated by using 2-way ANOVA for overall significance, followed by the Tukey-Kramer test. Statistical significance was defined as *P* < 0.05.

### Study approval.

All animal experiments were reviewed and approved by the IACUC of Brown University. Utilization of human lung samples was done in compliance with the IRB guidelines of Women and Infants Hospital.

## Author contributions

HY designed the study, conducted experiments, acquired and analyzed data, drafted the initial manuscript, and revised the manuscript. JG, ZF, ALP, JFC, XL, HZ, XJ, YYZ, and MEDP performed experiments and data analysis. PAD assisted with the experimental design and revised the manuscript. All authors approved the final manuscript as submitted.

## Supplementary Material

Supplemental data

## Figures and Tables

**Table 1:**
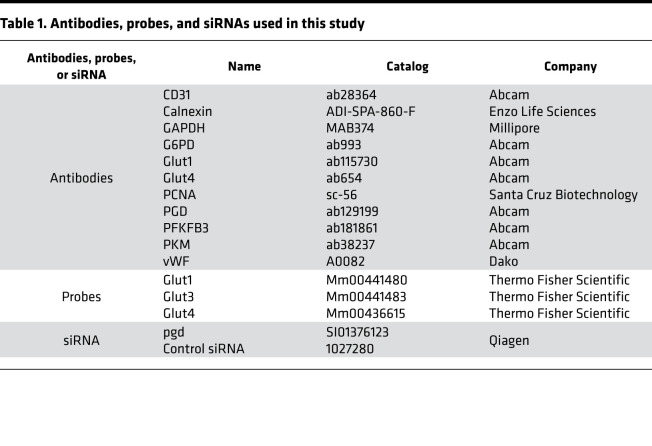
Antibodies, probes, and siRNAs used in this study

## Version 1 01/26/2021

In-Press Preview

## Version 2 03/08/2021

Electronic publication
