# Supplementary material for: The pentose phosphate pathway mediates hyperoxia-induced lung vascular dysgenesis and alveolar simplification in neonates
Source: JCI Insight. 2021 Mar 8;6(5):e137594. doi: 10.1172/jci.insight.137594 (PMC8021105; doi:10.1172/jci.insight.137594)
Supplement: Supplemental data [file jciinsight-6-137594-s147.pdf]

Supplemental Material

The pentose phosphate pathway mediates hyperoxia-induced lung vascular dysgenesis and alveolar simplification in neonates

Jiannan Gong[1,2], Zihang Feng[1], Abigail L. Peterson[1], Jennifer F. Carr[1], Xuexin Lu[1], Haifeng Zhao[1], Xiangming Ji[3], You-Yang Zhao[4,5], Monique E. De Paepe[6], Phyllis A. Dennery[1,7], Hongwei Yao[1]

[1]Department of Molecular Biology, Cell Biology & Biochemistry, Division of Biology and Medicine, Brown University, Providence, RI, USA;
[2]Department of Respiratory Medicine, Second Hospital of Shanxi Medical University, Shanxi Medical University, Taiyuan, Shanxi, China;
[3]Department of Nutrition, Byrdine F. Lewis School of Nursing and Health Professions, Georgia State University, Atlanta, GA, USA;
[4]Program for Lung and Vascular Biology, Stanley Manne Children's Research Institute, Ann & Robert H. Lurie Children's Hospital of Chicago, Chicago, Illinois, USA;
[5]Departments of Pediatrics (Critical Care Division), Pharmacology, and Medicine (Pulmonary and Critical Care Medicine), Northwestern University Feinberg School of Medicine, Chicago, Illinois, USA;
[6]Department of Pathology, Women and Infants Hospital, Providence, RI, USA;
[7]Department of Pediatrics, Warren Alpert Medical School of Brown University, Providence, RI, USA

To whom correspondence should be addressed:
Hongwei Yao, PhD,
Department of Molecular Biology, Cell Biology & Biochemistry,
Division of Biology and Medicine, Brown University,
185 Meeting Street, SFH,
Providence, RI, 02912, USA.
Phone: 401-863-6754;
Email: Hongwei_Yao@brown.edu

Conflict of interest: JG, ZF, ALP, JFC, XL, HZ, XJ, MED, PAD, and HY have declared that no conflict of interest exists. YYZ is the founder and Chief Scientific Officer of MountView Therapeutics, LLC, and is an inventor of a pending patent PCT/US2019/055787.

Supplemental Figure 1

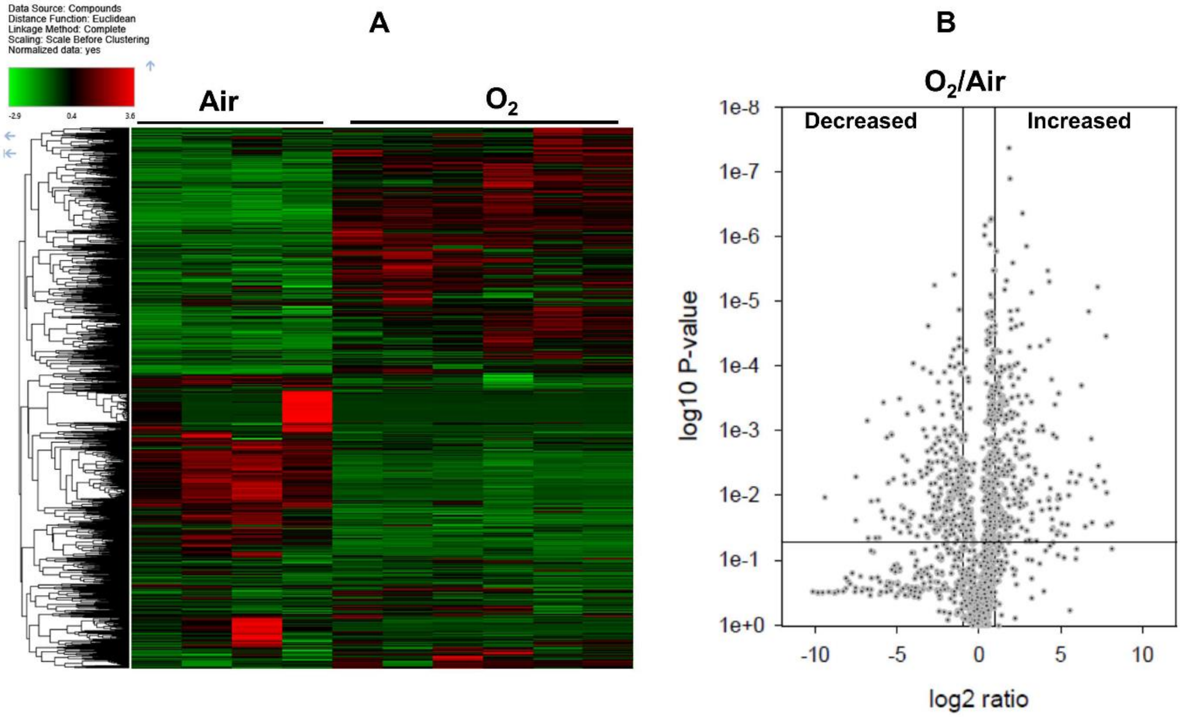

Supplemental Figure 1. Heatmap and volcano plot of untargeted metabolomics analysis in cultured lung ECs. Primary mouse LMVECs were exposed to hyperoxia for 24 h followed by air recovery for 24 h (refers to O2). Untargeted metabolomics analysis was performed by mass spectrometry. (A) Overall clustering for the correlation between air and hyperoxia groups. Scale is based on colors from green to red representing negative and positive correlations, respectively. (B) The -log10 *t*-test *P* values were plotted against the log2 ratio as a volcano plot of 1383 metabolic features detected in cells exposed to hyperoxia after normalization into air group. Each metabolite is represented as a dot. The upper right and upper left areas of this plot represent 2-fold increase or decrease, respectively, in hyperoxia-exposed cells compared to the air group ($P<0.05$). N=4 in air and N=6 in hyperoxia.

Supplemental Figure 2

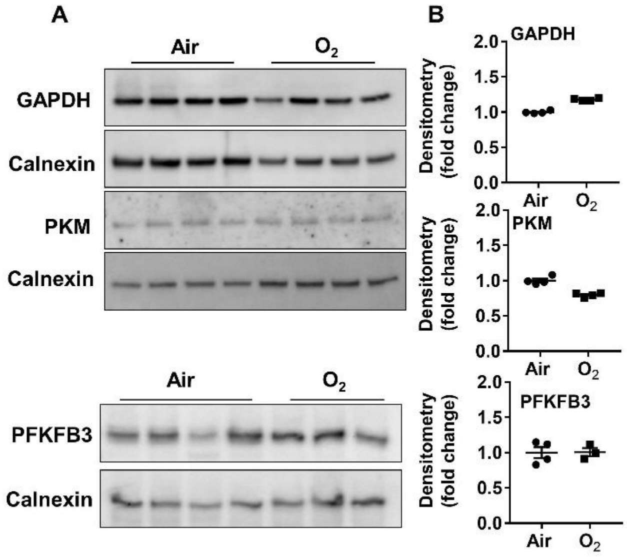

Supplemental Figure 2. Protein levels of key glycolytic enzymes in cultured lung ECs. Primary mouse LMVECs were exposed to hyperoxia for 24 h followed by air recovery for 24 h (refers to O2). Western blot was performed to determine the levels of GAPDH, PKM, and PFKFB3 proteins. Data are expressed as mean ± SEM. N=4 per group. The t-test was used for detecting statistical differences (A and B).

Supplemental Figure 3

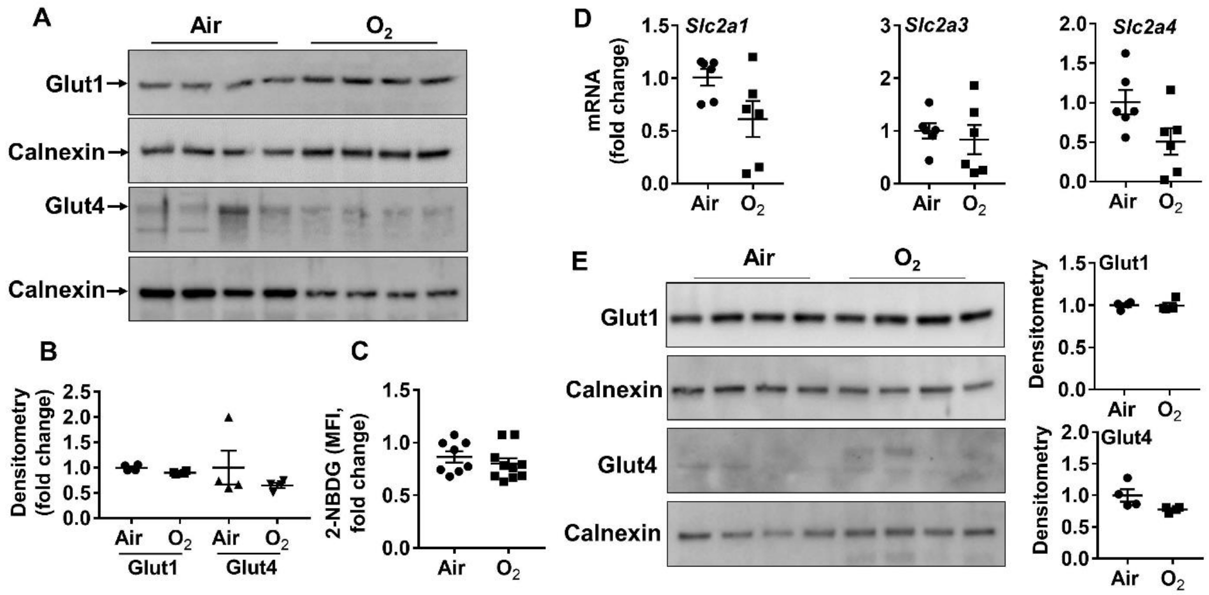

Supplemental Figure 3. Hyperoxic exposure did not affect glucose uptake in cultured lung ECs. MFLM-91U cells were exposed to hyperoxia for 24 h followed by air recovery for 24 h (refers to O2). (A and B) Levels of glut1 and glut4 proteins were detected using Western blot. N=4 per group. (C) 2-NBDG uptake was measured by flow cytometry. N=8 in air and N=10 in hyperoxia. C57BL/6J neonatal mice (<12 h old) were exposed to air or hyperoxia (95% O_2) for 3 days, and then allowed for recover in room air until pnd14. (D) mRNA and (E) protein levels of glut1 and glut4 in mouse lungs were determined by qRT-PCR and Western blot, respectively. N=6 per group (D) and N=4 per group (E). Data are expressed as mean ± SEM. The t-test was used for detecting statistical differences (A-E).

Supplemental Figure 4

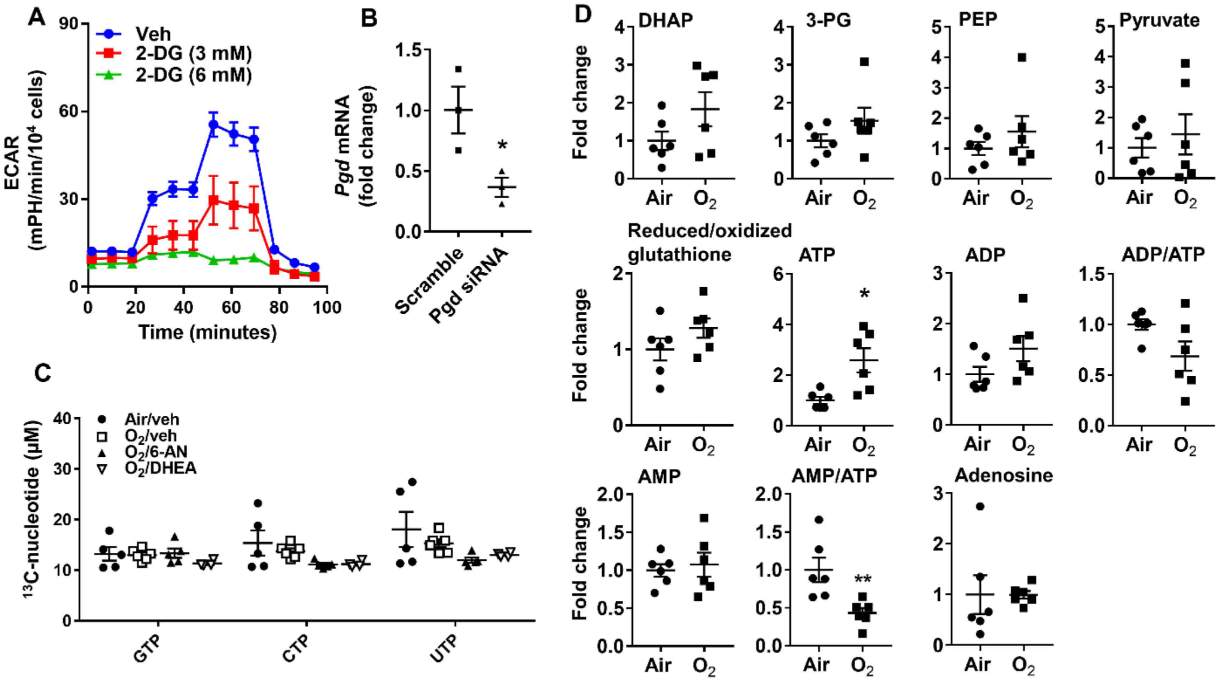

Supplemental Figure 4. 2-DG decreases glycolysis in cultured lung ECs, and hyperoxic exposure influences glucose-derived nucleotides in cells and ATP levels in mouse lungs. (A) ECAR was measured by the Seahorse Analyzer after 2-DG incubation (3 and 6 mM, 12 h) during air recovery phase in primary LMVECs. N=8 per group. (B) MFLM-91 cells were transfected with scramble or *pgd* siRNA for 48 h. Gene expression of *pgd* was measured by qRT-PCR. N=3 per group. (C) MFLM-91U cells were exposed to hyperoxia for 24 h followed by air recovery for 24 h (refers to O_2). ^{13}C-labled NTPs were measured by mass spectrometry when cells were incubated with 20 mM U-^{13}C-glucose for 24 h during air recovery phase. N=5 per group. (D) C57BL/6J neonatal mice (<12 h old) were exposed to air or hyperoxia (95% O_2) for 3 days, and then allowed for recover in room air until pnd7. Untargeted metabolomics was performed by mass spectrometry in mouse lungs. Levels of intracellular metabolites were present. N=6 per group. Data are expressed as mean ± SEM. $^{*}P<0.05$, $^{**}P<0.01$ vs scramble siRNA (B) or air (D) using t-test (B and D) or ANOVA followed by Tukey-Kramer test (C).

Supplemental Figure 5

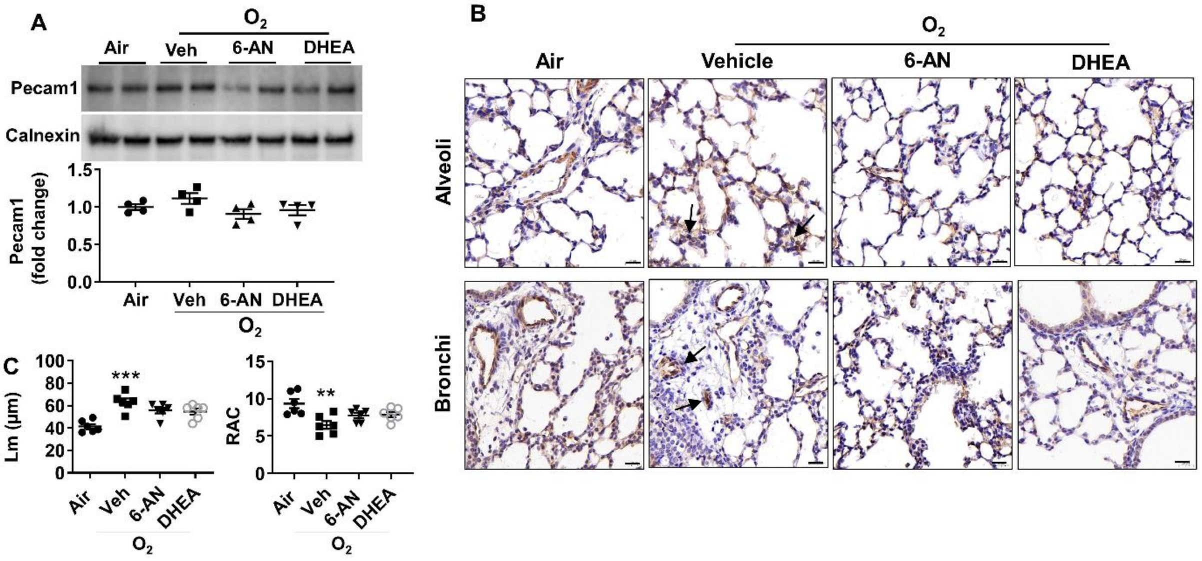

Supplemental Figure 5. Hyperoxic exposure causes dysmorphic lung vascular development in neonatal mice. C57BL/6J neonatal mice (<12 h old) were exposed to air or hyperoxia (95% O_2) for 3 days, and then allowed for recover in room air until pnd14. 6-AN (10 mg/kg, i.p.) or DHEA (20 mg/kg, i.p.) were administered daily in mice between pnd9 and pnd13 (A, B) or between pnd12 and pnd13 (C). (A) Western blot was performed to determine the levels of CD31 in mouse lungs. N=4 per group. (B) Immunohistochemistry was performed to assess CD31 abundance in mouse lungs. Representative images are present. Arrows denote CD31 positive cells. Bar size: 20 μm. (C) Mean linear intercept (Lm) and radial alveolar count (RAC) were measured in mouse lungs. N=6 per group. Data are expressed as mean ± SEM. $^{**}P<0.01$, $^{***}P<0.001$ vs air using ANOVA followed by Tukey-Kramer test (A-C).
